# MnCo_2_S_4_‐CoS_1.097_ Heterostructure Nanotubes as High Efficiency Cathode Catalysts for Stable and Long‐Life Lithium‐Oxygen Batteries Under High Current Conditions

**DOI:** 10.1002/advs.202103302

**Published:** 2021-10-18

**Authors:** Qing Xia, Lanling Zhao, Zhijia Zhang, Jun Wang, Deyuan Li, Xue Han, Zhaorui Zhou, Yuxin Long, Feng Dang, Yiming Zhang, Shulei Chou

**Affiliations:** ^1^ Key Laboratory for Liquid‐Solid Structural Evolution and Processing of Materials (Ministry of Education) Shandong University Jinan 250061 China; ^2^ Institute for Carbon Neutralization College of Chemistry and Materials Engineering Wenzhou University Wenzhou 325035 China; ^3^ School of Physics Shandong University Jinan 250100 P. R. China; ^4^ School of Materials Science and Engineering Tiangong University Tianjin 300387 China

**Keywords:** cathode catalysts, electrocatalysis, heterostructure, Li‐O_2_ batteries, MnCo_2_S_4_‐CoS_1.097_

## Abstract

Constructing the heterostructures is considered to be one of the most effective methods to improve the poor electrical conductivity and insufficient electrocatalytic properties of metal sulfide catalysts. In this work, MnCo_2_S_4_‐CoS_1.097_ nanotubes are successfully prepared via a reflux‐ hydrothermal process. This novel cathode catalyst delivers high discharge/charge specific capacities of 21 765/21 746 mAh g^−1^ at 200 mA g^−1^ and good rate capability. In addition, a favorable cycling stability with a fixed specific capacity of 1000 mAh g^−1^ at high current density of 1000 mA g^−1^ (167 cycles) and 2000 mA g^−1^ (57 cycles) are delivered. It is proposed that fast transmission of ions and electrons accelerated by the built‐in electric field, multiple active sites from the heterostructure, and nanotube architecture with large specific surface area are responsible for the superior electrochemical performance. To some extent, the rational design of this heterostructured metal sulfide catalyst provides guidance for the development of the stable and efficient cathode catalysts for Li‐O_2_ batteries that can be employed under high current conditions.

## Graphical abstract

MnCo_2_S_4_‐CoS_1.097_ nanotubes with rich hetero‐interfaces expose abundant active sites and promote the transport of electrons and ions, thus delivering superior cycling performance at high currents for Li‐O_2_ batteries.

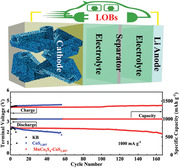

## 1 Introduction

Lithium‐oxygen batteries (LOBs) are expected to be used in the next generation of power sources due to their ultrahigh energy density (3500 Wh kg^−1^),^[^ ^1^, ^2^, ^3^ ^]^ but there are still some unsolved problems, such as poor cycle stability, inferior rate performance, and high charge/discharge overpotentials.^[^ ^4^, ^5^, ^6^ ^]^ Researchers found that one of the main reasons for the appeal challenge is the slow reaction kinetics in the process of oxygen reduction reaction (ORR) and oxygen evolution reaction (OER). Therefore, there is an urgent need for high‐performance cathode catalysts to promote the electrocatalytic activities.^[^ ^7^, ^8^, ^9^ ^]^

Precious metals have been found to serve as ideal electrochemical cathode catalyst to alleviate polarization and improve LOB performance,^[^ ^10^, ^11^ ^]^ but due to the scarcity and high price have limited their practical application, various alternatives were extensively investigated in LOBs. Such as transition metal oxides,^[^ ^12^, ^13^, ^14^ ^]^ sulfides,^[^ ^15^, ^16^, ^17^ ^]^ nitrides,^[^ ^18^, ^19^ ^]^ carbides,^[^ ^20^, ^21^ ^]^ carbon composites,^[^ ^22^, ^23^, ^24^ ^]^ and alloys.^[^ ^25^, ^26^ ^]^ Among them, metal sulfides have attracted extensive attention of researchers, because they own the following advantages: 1) Metal sulfides are normally inherently unstable, and various crystal defects thus form during the formation process, which is conducive to the production of abundant active sites.^[^ ^27^ ^]^ 2) Variable valences of metal cations could promote the catalytic reactions.^[^ ^28^ ^]^ However, the metal sulfides catalyst still has some problems that need to be solved urgently. In particular, metal sulfides are usually semiconductors or insulators,^[^ ^29^ ^]^ and their low electrical conductivity seriously restrain the reaction kinetics in the ORR/OER processes. Based on this fact, a variety of methods have been used to improve the electrical conductivity of metal sulfides to further exert their catalytic activity, mainly including introducing heterogeneous atoms and adopting carbon matrix materials.^[^ ^30^, ^31^ ^]^ However, the doping of heterogeneous atoms usually results in the formation of other substances in the metal sulfides, deteriorating electrocatalytic behaviors. In addition, carbon materials have been involved in unavoidable side reactions with superoxide, leading to a rapid increase in charging overpotentials. In recent years, heterostructure catalysts have developed rapidly, especially in the fields of hydrogen evolution reaction (HER),^[^ ^32^ ^]^ photocatalysis,^[^ ^33^ ^]^ water splitting,^[^ ^34^ ^]^ etc. Nowadays, Heterostructures can be defined as the unique architecture that consists of hetero‐interfaces formed by different solid‐state materials through physical and chemical combinations. Due to the difference in Fermi energy levels between different materials, the electrons transfer across the hetero‐interface from material with high Fermi energy levels to that with lower ones, thereby generating an equilibrium state of equal Fermi energy levels. In order to balance their original electron affinity (*ɸ*) and work function (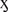) constant, a space charge region would be formed. Built‐in field plants will appear on both sides of the heterogeneous interface, which can greatly accelerate the transportation of electrons and ions, thereby remarkably boosting the electrocatalytic properties. It is also evident that constructing a heterostructure can also provide more active sites by introducing disordered atomic arrangement.^[^ ^35^, ^36^, ^37^ ^]^

Herein, we have successfully prepared MnCo_2_S_4_‐CoS_1.097_ heterostructure nanotubes by reflux and hydrothermal method. Benefitting from unique structure and architecture with large surface area, enough space could be provided for storing discharge products, and rich heterogeneous interfaces were constructed to promote fast transport of oxygen, ions, and electrons, endowing MnCo_2_S_4_‐CoS_1.097_ cathodes large specific capacities, good rate performance, and excellent cycling stability.

## 2 Results and Discussion

**Scheme 1:**
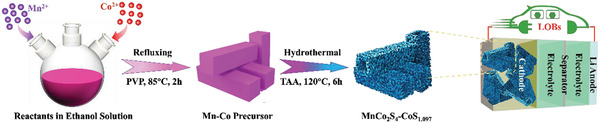
Schematic illustration for the preparation process of MnCo_2_S_4_‐CoS_1.097_ heterostructure nanotubes.

MnCo_2_S_4_‐CoS_1.097_ was synthesized by a reflux‐ hydrothermal process as shown in **Scheme** **1**. In the typical synthesis route, Mn(OAc)_2_ 4H_2_O, Co(OAc)_2_ 4H_2_O and polyvinyl pyrrolidone were uniformly dispersed and mixed in ethanol under magnetic stirring, and Mn‐Co precursor was achieved after refluxing treatment. Afterward, MnCo_2_S_4_‐CoS_1.097_ was achieved via hydrothermal reaction with the addition of thioacetamide (TAA).

**Figure 1:**
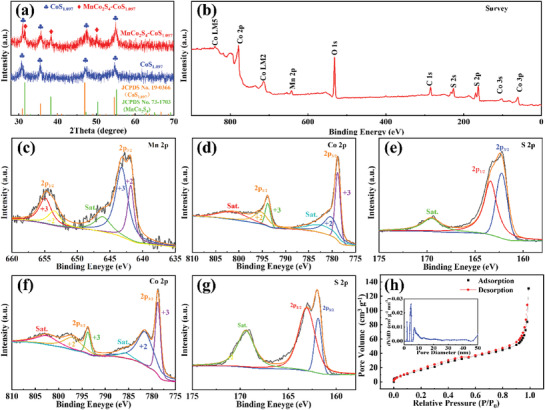
a) XRD patterns of different samples; b) XPS survey spectrum of MnCo_2_S_4_‐CoS_1.097_; high‐resolution XPS spectra of c) Mn 2p, d) Co 2p, and e) S 2p of MnCo_2_S_4_‐CoS_1.097_; high‐resolution XPS spectra of f) Co 2p and g) S 2p of CoS_1.097_; h) the nitrogen adsorption–desorption isotherms and pore size distribution curve of MnCo_2_S_4_‐CoS_1.097_.

In order to investigate the crystal structure and composition of the prepared material, the samples were analyzed and characterized by XRD. **Figure** **1**a shows the XRD patterns of the as‐prepared MnCo_2_S_4_‐CoS_1.097_ and CoS_1.097_ samples. The peaks at 30.9°, 35.5°, 47.1°, and 54.7° can be assigned to (204), (220), (306), and (330) faces of CoS_1.097_ (JCPDS No.19‐0366), indicate that the CoS_1.097_ sample is composed of *β*‐CoS_1.097_ phase with a hexagonal structure in the space group P63/mmc (194), while in the MnCo_2_S_4_‐CoS_1.097_ XRD spectrum, the extra peaks at 31.5°, 38.2°, and 50.4° are associated with the (311), (400), and (511) faces of MnCo_2_S_4_ (JCPDS No.73‐1703), which is similar to the previous result.^[^ ^38^ ^]^

X‐ray photoelectron spectroscopy (XPS) measurement was performed to further study the chemical composition and elemental chemical state of the samples. Survey spectra of MnCo_2_S_4_‐CoS_1.097_ and CoS_1.097_ are shown in Figure 1b and Figure S1, Supporting Information. For MnCo_2_S_4_‐CoS_1.097_, the characteristic peaks of the Co, Mn, and S can be detected, while it is found that the CoS_1.097_ sample is composed of only Co and S elements. In Figure 1c of the high‐resolution spectrum of Mn 2p, these peaks are in good agreement with two spin‐orbit doublets and a shaking satellite peak. The fitted peaks with binding energies of 641.8 and 653.5 eV are related to Mn^2+^ signals, and the fitted peaks with binding energies of 643.2 and 655.0 eV are ascribed to the Mn^3+^ signals, respectively. By integrating the areas under different peaks, the corresponding Mn^3+^/Mn^2+^ content can be calculated to be 68%/32%. In a similar way, Co 2p can be fitted into two spin‐orbit doublets (Figure 1d,f). The fitted peaks of Co^2+^ signals locate at 780.4 and 795.5 eV, while those of Co^3+^ signals locate at 778.8 and 793.8 eV, respectively. The ratios of Co^3+^/Co^2+^ on MnCo_2_S_4_‐CoS_1.097_ and CoS_1.097_ surfaces were determined to be 46/54 and 38%/62%, respectively. These results show that in the MnCo_2_S_4_‐CoS_1.097_ and CoS_1.097_ samples, both valence states of Mn and Co are divalent and trivalent, which could serve as effective multiple active sites for electrocatalytic reactions. The spectrum of S2p (Figure 1e) shows three main peaks. The fitted peaks at 162.3 and 163.5 eV are assigned to S 2p_1/2_ and S 2p_3/2_, while the peak at 169.4 eV is the typical characteristic of sulfur ions with metal ions, respectively. In order to further determine the chemical compositions of the samples, the inductively coupled plasma measurements were performed on the MnCo_2_S_4_‐CoS_1.097_, and CoS_1.097_ samples, and the contents of each element are as shown in Figure S2, Supporting Information. For MnCo_2_S_4_‐CoS_1.097_, the ratio of Mn/Co/S is 1/3.19/5.34. Through further calculation, it can be concluded that the proportions of MnCo_2_S_4_ and CoS_1.097_ in the composite are 45.3% and 54.7%, respectively. For CoS_1.097_, the ratio of Co/S is 1/1.11, which is well consistent with the theoretical value. The N_2_ adsorption–desorption studies were also conducted for different samples, as given in Figure 1h and Figure S3, Supporting Information. It is clear that the N_2_ adsorption isotherms of MnCo_2_S_4_‐CoS_1.097_ present IV type H3 hysteresis loop with the *P*/*P*_0_ ranging from 0.2 to 1.0, and the hysteresis loop exhibits a saturated adsorption platform, indicating uniform pore formation. The specific surface areas of MnCo_2_S_4_‐CoS_1.097_ and CoS_1.097_ are 57.8001 and 6.3240 m^2^ g^−1^, and their total pore volume are 0.24 and 0.012 cm^3^ g^−1^, respectively, which demonstrates that MnCo_2_S_4_‐CoS_1.097_ is more conducive to the mass transfer and efficient storage of discharge products (Li_2_O_2_).

**Figure 2:**
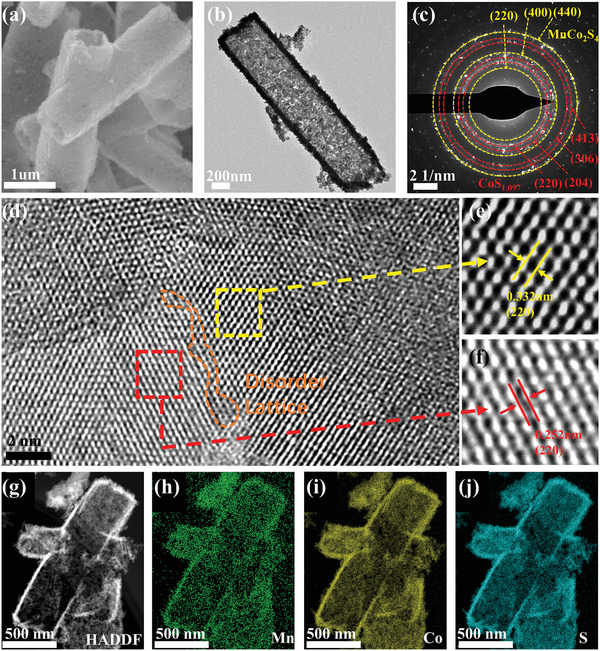
a) SEM image of the MnCo_2_S_4_‐CoS_1.097_; b) TEM image of the MnCo_2_S_4_‐CoS_1.097_; c) SAED pattern of the MnCo_2_S_4_‐CoS_1.097_; d–f) HRTEM images of the MnCo_2_S_4_‐CoS_1.097_; g–j) elemental mapping images of MnCo_2_S_4_‐CoS_1.097_.

Morphology of precursor, MnCo_2_S_4_‐CoS_1.097_ and CoS_1.097_ were captured by field emission scanning electron microscope, and the data are shown in **Figure** **2**a and Figures S4,S5, Supporting Information. The precursor appears as a typical solid tubes‐like structure, and its height and bottom side length are about 2 and 0.8um, respectively. The tubes‐shaped structure of CoS_1.097_ is more rounded, and its height is only around 400 nm. The TEM image in Figure S6a, Supporting Information shows that the tube‐like structure is not hollow, and its cross section seems like ellipse shape. The high‐resolution transmission electron microscope(HRTEM) image in Figure S6c, Supporting Information and the intensity profile in Figure S6d, Supporting Information recorded from the corresponding region of CoS_1.097_ clearly show the (220) crystal lattice fringes with the spacing of 0.252 nm. Moreover, the pattern of selected area electron diffraction (SAED) of CoS_1.097_ in Figure S6b, Supporting Information indicates (204), (220), and (306) faces of CoS_1.097_. In contrast, MnCo_2_S_4_‐CoS_1.097_ shows a hollow tubes‐shaped structure. Its size is similar to that of the precursor, but the surfaces are rough and uneven, resulting in a larger surface area. Moreover, the hollow structure could enable more exposed active sites and abundant three‐phase reaction zones, which provide enough spaces for Li_2_O_2_ storage and diffusion tunnels for reactants (Li^+^, O_2_) to the reaction sites.

TEM was used to study the more detailed microstructure characteristics of MnCo_2_S_4_‐CoS_1.097_, as shown in Figure 2b, it can be seen that the inside of the tube is hollow, and the surface of the hollow tube is composed of particles with a scale of about 20 nm, which is highly consistent with the scanning electron microscope (SEM) image. As shown in Figure 2c, the diffraction ring in the electron diffraction (SAED) mode of MnCo_2_S_4_‐CoS_1.097_ can be respectively indexed as (220), (400), and (440) planes of MnCo_2_S_4_, and (220), (304), (306), and (413) planes of CoS_1.097_, which further proved the successful preparation of MnCo_2_S_4_‐CoS_1.097_ composites. HRTEM image of MnCo_2_S_4_‐CoS_1.097_ is shown in Figure 2d–f. The clearly defined lattice fringes of 0.332 nm in the Figure 2f can be clearly attributed to (220) plane of MnCo_2_S_4_, and the lattice fringe distance of 0.252 nm is attributed to the (220) crystal plane of CoS_1.097_. The interface area between MnCo_2_S_4_ and CoS_1.097_ species is shown in the orange area of Figure 2d. Under the strong electronic interaction between MnCo_2_S_4_ and CoS_1.097_, the crystal lattice in this region is distorted, which may lead to the increased catalytic sites with the unique electrochemical behaviors. The element mapping diagram of MnCo_2_S_4_‐CoS_1.097_ (Figure 2g–j) clearly reveals the uniform distribution of Mn, Co, and S elements, further verifying the successful preparation of unique hollow heterostructure of MnCo_2_S_4_‐CoS_1.097_.

**Figure 3:**
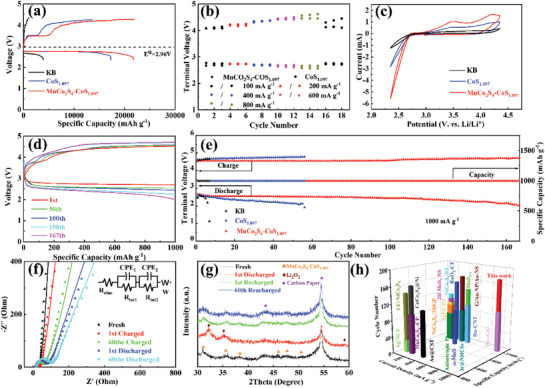
a) Initial discharge/charge profiles of different cathodes at 200 mA g^−1^ from 2.35 to 4.35 V; b) comparison of rate performance of different cathodes; c) CV curves of different cathodes at 0.15 mV s^−1^; d) typical discharge/charge profiles of MnCo_2_S_4_‐CoS_1.097_ cathode at 500 mA g^−1^; e) cycling performance of different cathodes at 1000 mA g^−1^ under a specific capacity limit of 1000 mAh g^−1^; f) EIS plots and g) XRD patterns of the MnCo_2_S_4_‐CoS_1.097_ cathodes at different stages; h) comparison of the cycling stability of MnCo_2_S_4_‐CoS_1.097_ cathode with those of representative and most recently reported cathodes based on metal sulfide catalysts and noble metals.

**Figure** **3**a shows the potential cut‐off constant current discharge–recharge voltage curves of CoS_1.097_, and MnCo_2_S_4_‐CoS_1.097_ cathodes for LOBs at a current density of 200 mA g^−1^. For better comparison, the specific capacity of KB and carbon paper cathode was also tested under the same conditions (Figure S7, Supporting Information). The CoS_1.097_ and MnCo_2_S_4_‐CoS_1.097_ cathodes show similar discharge voltages at 2.74 V, which are 0.12 V higher than that of the KB cathode. It is evident that MnCo_2_S_4_‐CoS_1.097_ and CoS_1.097_ cathodes show a two‐stage charging platform during the charging process. At around 3.5 V, the active sites on the cathode surface absorbed O_2_ to reduce it, on which the adsorbed oxygen (O_2_*) was combined with Li^+^ to form an adsorbed lithium superoxide (LiO_2_*) (Li^+^ + O_2_* + e^−^→LiO_2_*).^[^ ^39^, ^40^ ^]^ At 4.2 V, further reduction of LiO_2_* to form adsorbed Li_2_O_2_* (LiO_2_* + Li^+^+ e^−^→Li_2_O_2_*) was conducted. It is found that the discharge/charge capacities of the MnCo_2_S_4_‐CoS_1.097_ and CoS_1.097_ cathodes are 21 765/21 746 and 17 302/13 626 mAh g^−1^, respectively, which are much higher than those of KB cathodes with lower overpotentials. The coulombic efficiencies of KB and CoS_1.097_ cathodes are 26% and 79%, while the MnCo_2_S_4_‐CoS_1.097_ cathode exhibits the coulombic efficiency of 99%. More importantly, the charging voltage of the MnCo_2_S_4_‐CoS_1.097_ cathodes is obviously less than that of the CoS_1.097_ cathodes. This indicates that the MnCo_2_S_4_‐CoS_1.097_ could deliver superior OER activity.

The battery tested sequentially at current densities ranging from 100 to 800 mA g^−1^ for each 3 cycles under the specific capacity limit of 1000 mAh g^−1^. It is clear in Figure 3b that the charge and discharge overpotentials of the two cathodes increased with the increase of the current densities. At each current density, MnCo_2_S_4_‐CoS_1.097_ cathode shows lower overpotentials in Figure S8, Supporting Information, which also demonstrated that MnCo_2_S_4_‐CoS_1.097_ cathode feature better ORR and OER properties. When the current density was restored to 100 mA g^−1^, the discharge and charge terminal voltages of the MnCo_2_S_4_‐CoS_1.097_ battery dropped sharply, with almost no change compared with that at the initial state. It is much more stable than the CoS_1.097_ counterparts, implying that the MnCo_2_S_4_‐CoS_1.097_ cathode hold excellent rate capability.

Cyclic voltammetry (CV) curves of three different cathodes were tested at a scan rate of 0.15 mV s^−1^ (Figure 3c). The first cycle curve shows that the MnCo_2_S_4_‐CoS_1.097_ cathode offers a higher current than that of the CoS_1.097_ and KB cathodes. In addition, unlike KB cathode, both the MnCo_2_S_4_‐CoS_1.097_ and CoS_1.097_ cathodes display oxidation peaks around 3.5 and 4.1 V, which is consistent with the charging platforms in Figure 3a. Moreover, the MnCo_2_S_4_‐CoS_1.097_ cathode shows the lower OER and higher ORR onset potentials, which also indicates that the MnCo_2_S_4_‐CoS_1.097_ cathode own better OER/ORR electrocatalytic properties. The reason for these result may be that the formation of a heterostructure could increases the electrical conductivity of the material, while the hollow architecture with a layered construction reduced the diffusion path length of ions and oxygen, thereby promoting electrocatalytic activity.

Figure 3d and Figure S9, Supporting Information show typical voltage curves of the MnCo_2_S_4_‐CoS_1.097_, CoS_1.097_, and KB cathodes during cycling at 1000 mA g^−1^ with a fixed capacity of 1000 mAh g^−1^. Figure 3e gives the discharge/charge terminal voltage curve diagram of three different cathodes, in which the MnCo_2_S_4_‐CoS_1.097_ cathode cycled 167 cycles at 2.0 to 5.0 V, while the CoS_1.097_ and KB cathodes only cycled 56 cycles and 7 cycles under the same conditions. It is worth noting that the ORR and OER overpotentials of the MnCo_2_S_4_‐CoS_1.097_ cathodes are significantly lower than that of the CoS_1.097_ and the KB cathodes. In addition, the cycle performance MnCo_2_S_4_‐CoS_1.097_ cathodes under the current conditions of 2000 mA g^−1^ with cut‐off specific capacity of 1000 mAh g^−1^ were also tested (Figure S10, Supporting Information), and cycled 57 times. This illustrates that the MnCo_2_S_4_‐CoS_1.097_ cathodes could exhibit superior stability and reversibility at high current densities.

Figure 3g shows the XRD pattern after 1st discharging. Two new peaks appeared at 32.7° and 34.8°, respectively, which corresponding to the (200) and (201) crystal planes (JCPDS#73)‐1640) of Li_2_O_2_.^[^ ^41^, ^42^ ^]^ In addition, SEM and TEM investigations have been conducted to recognize the discharge products. As shown in Figure S11a,c, Supporting Information, the dense package products precipitated on the surfaces and filled the center hole of the MnCo_2_S_4_‐CoS_1.097_ cathodes after discharging. HRTEM image in Figure S11d, Supporting Information shows a spacing of 0.258 nm, corresponding to the (201) lattice of Li_2_O_2_, and the different lattice fringes in the SAED pattern in Figure S11b, Supporting Information well coincided with the crystal lattices of MnCo_2_S_4_, CoS_1.097_, and Li_2_O_2_. After 1st and 60th recharging, the XRD pattern rarely changed compared at its initial state, which confirms the stable cycle performance of the MnCo_2_S_4_‐CoS_1.097_ cathodes. Electrochemical impedance spectroscopy (EIS) data at different stages are shown in Figure 3f with the corresponding equivalent circuit in the inset. Typically, *R*_ohm_ represents the Ohmic resistance of the Li‐O_2_ cell, and *R*_int1_ and *R*_int2_ denote the charge transfer resistance between the cathode/electrolyte and Li anode/electrolyte, respectively. The mass diffusion rate is related to the speed of transporting reaction species. The variations of *R*_int1_ during cycling are associated with the deposition of discharge products.^[^ ^22^, ^43^ ^]^ The sudden increase of the *R*_int1_ from 13.5 Ω at fresh stage to 45.9 Ω after discharging is due to the covering of insulating Li_2_O_2_ on cathode surfaces, which normally decreased the electrical conductivity and increased the charge transfer resistance.^[^ ^44^, ^45^ ^]^ After 1st charging, Li_2_O_2_ decomposed, and the *R*_int1_ became 18.7 Ω, almost equal to that at the pristine state. After 60th charging, the charge transfer resistance slightly increased, indicating the excellent cycling performance of MnCo_2_S_4_‐CoS_1.097_ cathodes. In addition, compared with the most of the reported typical sulfides^[^ ^46^, ^47^, ^48^, ^49^, ^50^, ^51^, ^52^, ^53^ ^]^ and noble metals based cathodes (Figure 3h),^[^ ^54^, ^55^, ^56^, ^57^, ^58^, ^59^, ^60^, ^61^, ^62^ ^]^ it is worth mentioning that the MnCo_2_S_4_‐CoS_1.097_ cathode deliver superior cycling stability under similar testing conditions.

**Figure 4:**
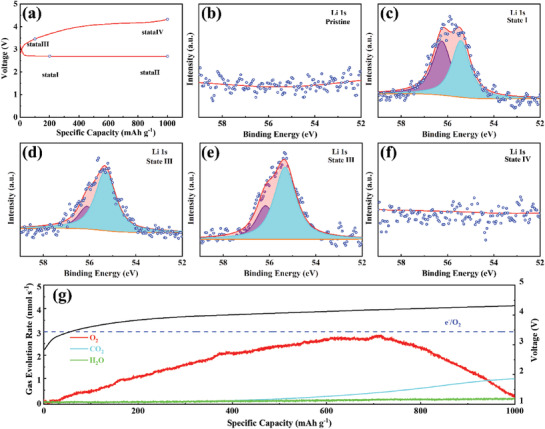
a) Discharge/charge curves for the initial cycle at 500 mA g^−1^ under a cutoff specific capacity of 1000 mAh g^−1^; b–f) high‐resolution Li 1s XPS spectra of MnCo_2_S_4_‐CoS_1.097_ cathodes at different states; g) in situ DEMS profiles for MnCo_2_S_4_‐CoS_1.097_ cathodes with charge curves at 500 mA g^−1^.

In order to reveal the discharging/charging mechanism during cycling based on MnCo_2_S_4_‐CoS_1.097_ cathodes, the high‐resolution XPS spectra of Li 1s at various stages (**Figure** **4**a) with limited specific capacity of 1000 mAh g^−1^ at 1000 mA g^−1^ were collected and shown in Figure 4c–f. According to previous literature reports, the binding energies at around 55.2 and 56.1 eV in Li 1s spectrum can be ascribed to the Li_2_O_2_ or Li_2 −_ *_x_* O_2_ (mixture of LiO_2_ and Li_2_O_2_), respectively.^[^ ^63^, ^64^, ^65^ ^]^ At State I, the proportion of Li_2 −_ *_x_* O_2_ and Li_2_O_2_ is 48%/52%, suggesting that in the initial stage of discharging, the following three reactions experienced at the same time:

(1) $$Li^{+}+O_{2(\mathrm{SOL})}+e^{-}\to \mathrm{Li}{O_{2}}^{*}$$

(2) $$Li^{+}+\mathrm{Li}{O_{2}}^{*}+e^{-}\to Li_{2}{O_{2}}^{*}$$

(3) $$2\mathrm{Li}{O_{2}}^{*}\to Li_{2}{O_{2}}^{*}+O_{2}$$

At State II, the ratio of Li_2 −_ *_x_* O_2_/Li_2_O_2_ becomes 24%/76%, indicating that more LiO_2_ was oxidized to Li_2_O_2_. At State III, the content of Li_2 −_ *_x_* O_2_ increased to 33%, indicating that at the beginning of the charging reaction, part of Li_2_O_2_ converted to LiO_2_ through the delithiation reaction. In order to confirm this conclusion, the gas evolution rates of O_2_, CO_2_, and H_2_O were tested by in situ differential electrochemical mass spectrometry (DEMS) with the galvanostatic charge voltage profile of MnCo_2_S_4_‐CoS_1.097_ presented in Figure 4g. The blue dotted line in the Figure 4g represents the amount of electrons passing per second (v(e^−^)), and the calculation result is 3.01 nmol s^−1^. When the charging specific capacity is 760 mAh g^−1^, the value of O_2_ release was approaching to the maximum, and only the decomposition of LiO_2_ happened. Before that, the ratio of the electron transfer rate to the release rate of oxygen v(e^−^):v(O_2_) is between 1 and 2, indicating that the decomposition of LiO_2_ and Li_2_O_2_ are simultaneously present in the subsequent charging process. During the initial charging process, the content of LiO_2_ increased, which indicates that Li_2_O_2_ decomposed according to the following steps, and the reaction rate of Equation (4) is faster, demonstrating the accumulation of LiO_2_.

(4) $$Li_{2}O_{2}\to \mathrm{Li}O_{2}+O_{2}+e^{-}$$

(5) $$\mathrm{Li}O_{2}\to Li^{+}+O_{2}+e^{-}$$

## 3 Conclusions

In summary, MnCo_2_S_4_‐CoS_1.097_ nanotubes were successfully constructed by simply reflux and hydrothermal process. The hollow structure could not only provide abundant active sites and sufficient storage room for discharge products, but also supply rich heterogeneous interfaces between the MnCo_2_S_4_ and CoS_1.097_ to promote fast transport of oxygen, ions, and electrons, thus exhibiting superior ORR/OER performance in LOBs. The MnCo_2_S_4_‐CoS_1.097_ nanotubes cathode shows high discharge/charge specific capacities of 21 765/21 746 mAh g^−1^ at 200 mA g^−1^, and it also delivers pleasant rate capability. In addition, outstanding cycling stability was also achieved with a retention capacity of 1000 mAh g^−1^ after 167 cycles at 1000 mA g^−1^ and 57 cycles at 2000 mA g^−1^. This rational synthetic strategy to construct heterogeneous structure could offer a guidance for the application of other sulfide materials in LOBs, which is also expected to be extended to other energy storage and conversion systems.

## 4 Experimental Section

### Preparation of the MnCo_2_S_4_‐CoS_1.097_ and CoS_1.097_

0.45 g of Mn(OAc)_2_⋅4H_2_O, 0.9 g of Co(OAc)_2_.4H_2_O, and 3.0 g of polyvinyl pyrrolidone (PVP, K15, Mw≈10 000) were dispersed in 200 mL ethanol, and a clear pink solution formed, which was then gradually heated to 85 °C and treated under reflux for 2 h. After cooling to room temperature, the resulting precipitate was collected by centrifugation and washed four times with ethanol and deionized water, followed by drying in an oven at 50 °C for 12 h to obtain the Mn‐Co precursor. Afterward, 0.08 g of the above‐mentioned Mn‐Co precursor and 0.120 g of TAA were added to 40 mL ethanol under stirring for 10 min. The solution was then transferred to an autoclave and kept at 120 °C for 6 h. After cooling down to room temperature, the product was collected by centrifugation, washed several times with ethanol and water, followed by drying in an oven at 60 °C for 12 h to gain MnCo_2_S_4_‐CoS_1.097_. CoS_1.097_ was achieved via the same procedure without adding Mn(OAc)_2_⋅4H_2_O.

### Material Characterizations

X‐ray diffractometer (RigakuD/ max 2500, Japan)) was used to study the phase composition. Morphology and elemental composition of the samples were analyzed by a field SEM (Hitachi, S‐4800, Japan) with energy dispersive X‐ray spectroscopy and a HRTEM (JEM‐ 2100F, 200 kV). The specific surface area and pore size distribution of the samples were determined by the N_2_ adsorption/desorption isotherms of Brunauer–Emmett–Teller with a Micrometritics analyzer (ASAP2020). An XPS (ESCALAB250) was performed to analyze the chemical species and bonding properties of MnCo_2_S_4_‐CoS_1.097_ and CoS_1.097_.

### Electrochemical Measurements

To fabricate the cathode, a slurry containing an active catalyst sample, KB and polytetrafluoroethylene with a mass ratio of 40:40:20 was uniformly dispersed in isopropanol with ultrasonic treatment. The mixture was then sprayed evenly on the carbon paper on a heating base to remove isopropanol. Then, it was held in a vacuum oven at 120 °C for 10 h to achieve the cathode. All batteries were assembled in a glove box (H_2_O < 0.1 ppm, O_2_ < 0.1 ppm) filled with an argon atmosphere. The batteries consisted of an as‐prepared cathode, a glass fiber separator, a Li metal foil as a counter anode, and the electrolyte of 1 m lithium bis(trifluoromethanesulfonyl)imide (LiTFSI) in tetraethylene glycol dimethyl ether. The Li‐O_2_ batteries were tested using a LAND test system (CTA2001A, Wuhan Land Electronics Co., Ltd.) in 2032 coin‐type cells in a high‐purity oxygen box with a cut‐off voltage from 2.35 to 4.35 V (relative to Li^+^/Li). The specific capacity and current were calculated based on the weight of the active catalyst supported on the cathode. CV was performed at a scan rate of 0.15 mV s^−1^ on an electrochemical workstation (PARSTAT MC2273), on which EIS were also conducted.

## Conflict of Interest

The authors declare no conflict of interest.

## Supporting information

Supporting Information

## Data Availability

The data that support the findings of this study are openly available in figshare at http://doi.org/10.1002/advs.202103302, reference number advs.202103302R1.
