# Supplementary material for: MnCo2S4‐CoS1.097 Heterostructure Nanotubes as High Efficiency Cathode Catalysts for Stable and Long‐Life Lithium‐Oxygen Batteries Under High Current Conditions
Source: Adv Sci (Weinh). 2021 Oct 18;8(22):2103302. doi: 10.1002/advs.202103302 (PMC8596117; doi:10.1002/advs.202103302)
Supplement: Supplementary file 1 — Supporting Information [file ADVS-8-2103302-s001.pdf]

WILEY-VCH

© 2021 Wiley-VCH GmbH

ADVANCED SCIENCE
Open Access

Supporting Information

for *Adv. Sci.*, DOI: 10.1002/advs.202103302

$MnCo_2S_4$-$CoS_{1.097}$ Heterostructure Nanotubes as High Efficiency Cathode Catalysts for Stable and Long-Life Lithium-Oxygen Batteries Under High Current Conditions

Qing Xia, Lanling Zhao, Zhijia Zhang, Jun Wang, Deyuan Li, Xue Han, Zhaorui Zhou, Yuxin Long, Feng Dang*, Yiming Zhang,* and *Shulei Chou**

Supporting Information

$MnCo_2S_4$-$CoS_{1.097}$ Heterostructure Nanotubes as High Efficiency Cathode Catalysts for Stable and Long-Life Lithium-Oxygen Batteries Under High Current Conditions

Qing Xia, Lanling Zhao, Zhijia Zhang, Jun Wang, Deyuan Li, Xue Han, Zhaorui Zhou, Yuxin Long, Feng Dang*, Yiming Zhang, Shulei Chou**

Q. Xia, Dr. J. Wang, D. Li, X. Han, Z. Zhao, Y. Long, Prof. F. Dang, Y. Zhang
Key Laboratory for Liquid-Solid Structural Evolution and Processing of Materials (Ministry of Education), Shandong University, Jinan 250061, China
Email: jw707@sdu.edu.cn; dangfeng@sdu.edu.cn
Dr. L. Zhao
School of Physics, Shandong University, Jinan 250100, P.R. China
Prof. Z. Zhang
School of Materials Science and Engineering, Tiangong University, Tianjin 300387, China
Prof. S. Chou
Institute for Carbon Neutralization, College of Chemistry and Materials Engineering, Wenzhou University, Wenzhou 325035, China
Email: chou@wzu.edu.cn

Keywords: $MnCo_2S_4$-$CoS_{1.097}$, Heterostructure, Cathode; Electrocatalysis, Li-O_2 batteries

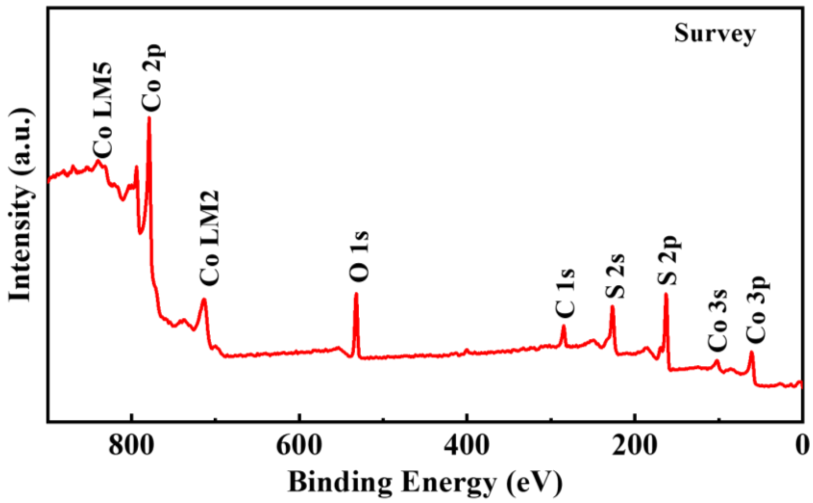

Figure S1. XPS survey spectra of $CoS_{1.097}$.

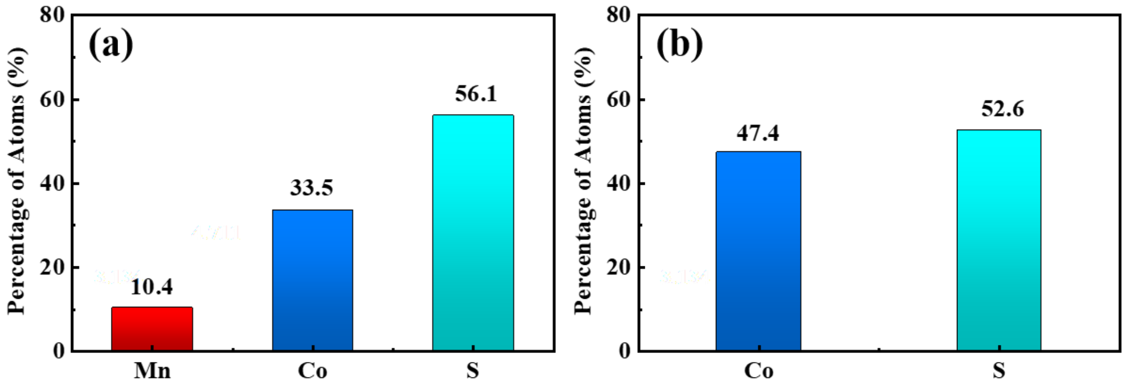

Figure. S2 Atom percentages of different atoms of (a) $MnCo_2S_4$-$CoS_{1.097}$ and (b) $CoS_{1.097}$.

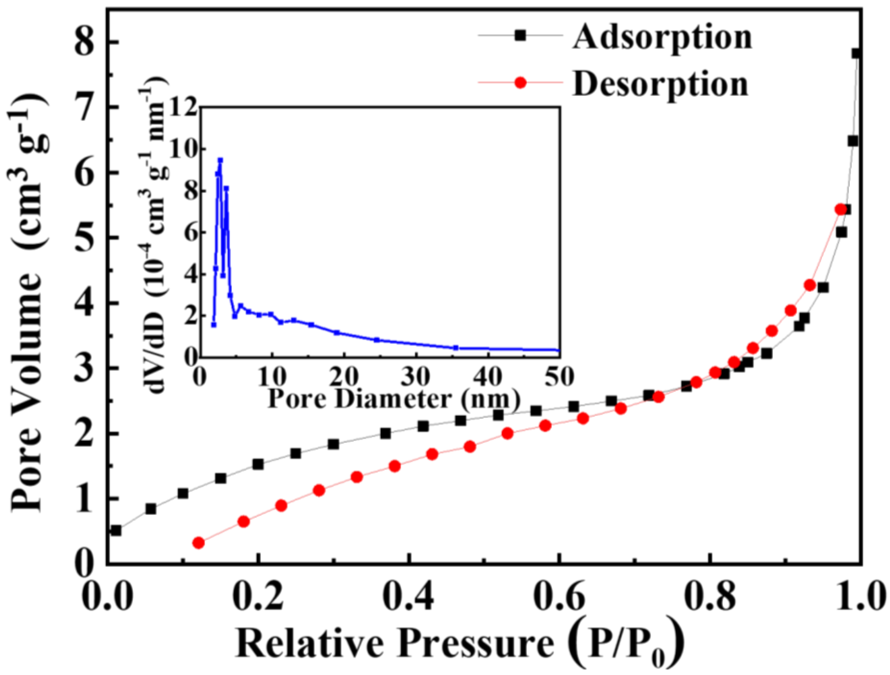

Figure S3. Nitrogen adsorption-desorption isotherms and pore size distribution curve of $CoS_{1.097}$.

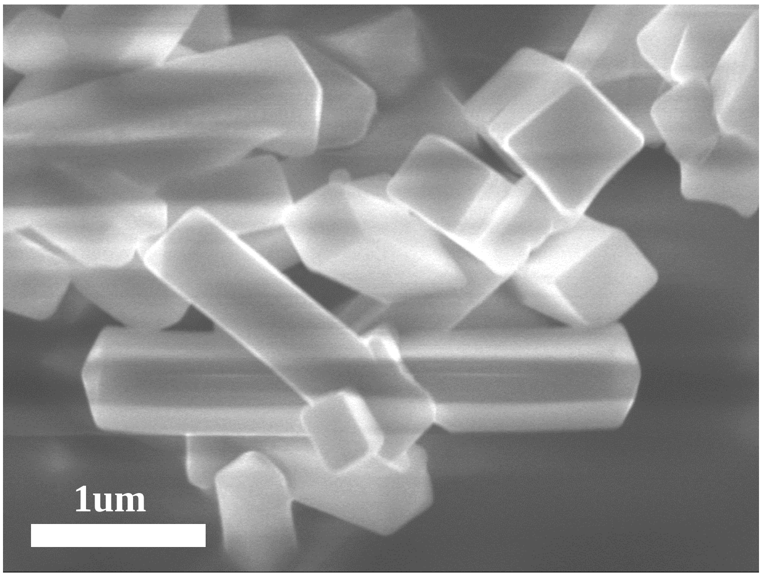

Figure S4. SEM image of precursor.

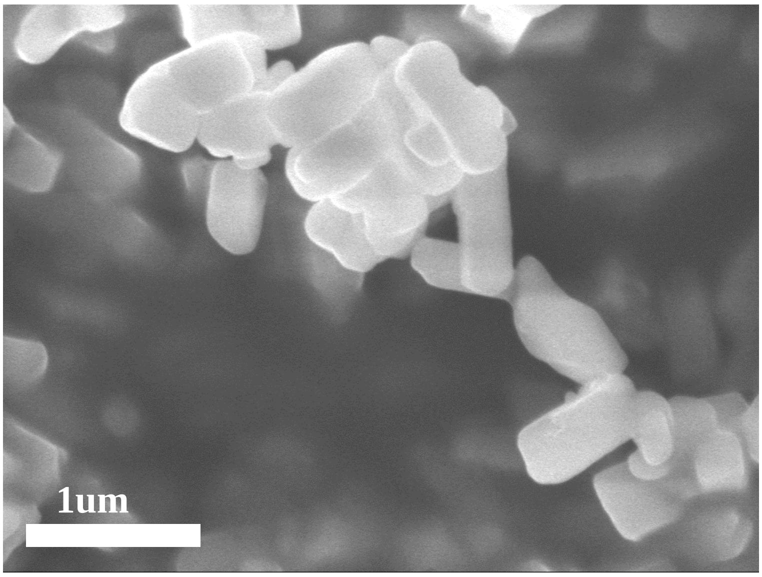

Figure S5. SEM image of $CoS_{1.097}$.

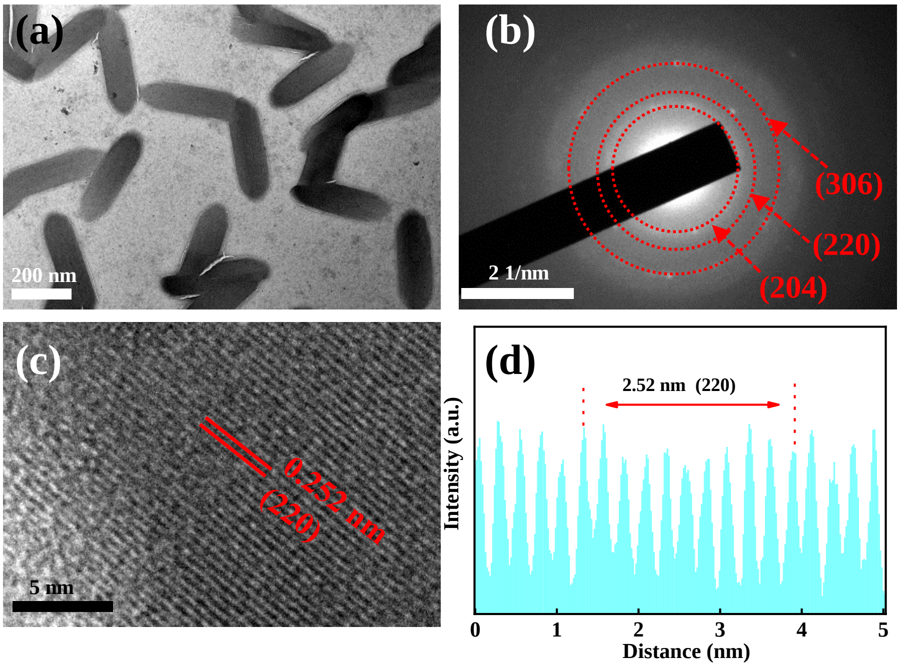

Figure S6. (a) TEM image, (b) SAED pattern and (c) HRTEM image with (d) intensity profile of $CoS_{1.097}$.

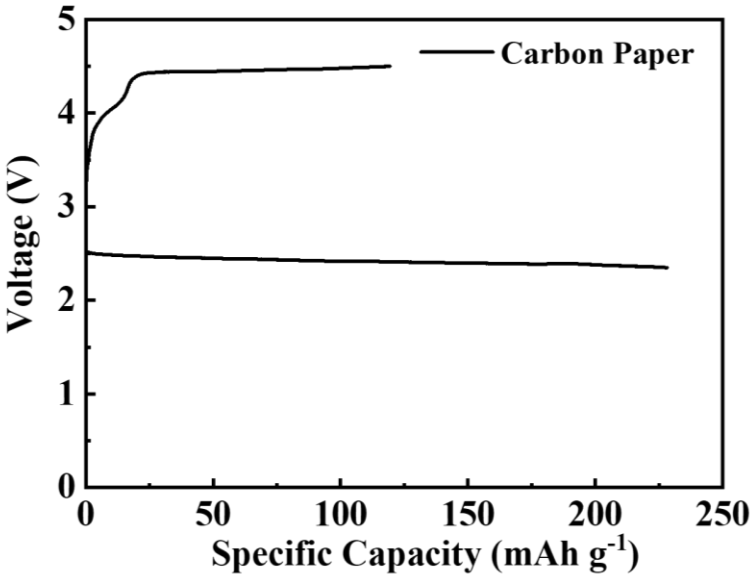

Figure S7. Initial discharge/charge profiles of carbon paper cathode at 200 mA g^{-1} from 2.35 to 4.5 V.

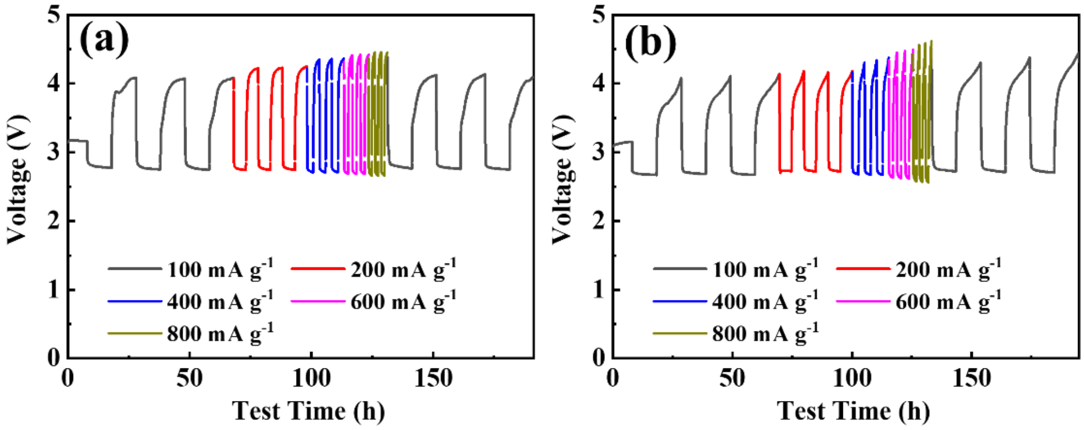

Figure S8. Rate performance of (a) $MnCo_2S_4$-$CoS_{1.097}$ and (b) $CoS_{1.097}$ cathodes.

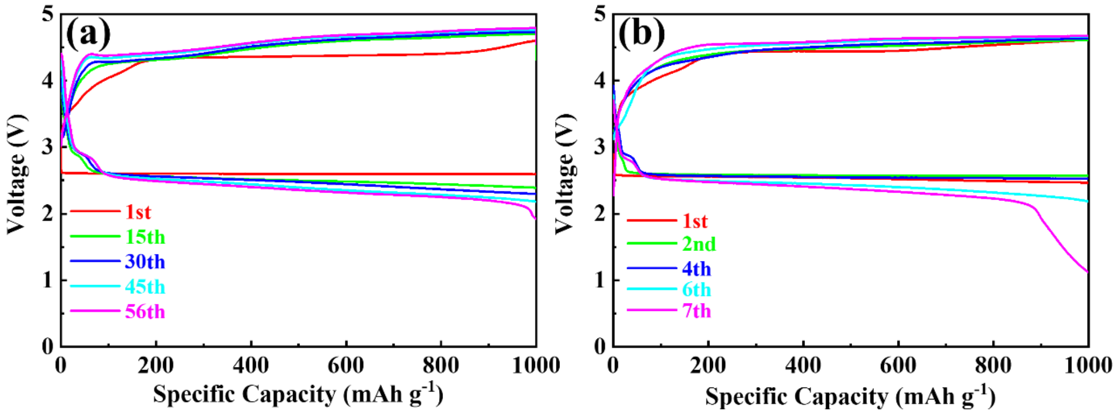

Figure S9. Typical discharge/charge profiles of (a) $MnCo_2S_4$-$CoS_{1.097}$ and (b) $CoS_{1.097}$ cathodes at 1000 mA g^{-1} with a cut-off specific capacity of 1000 mAh g^{-1}.

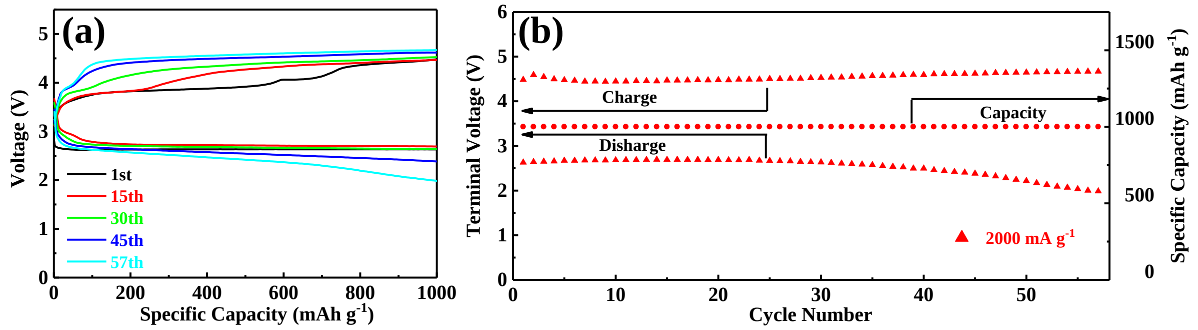

Figure S10. (a) Typical discharge/charge profiles of $MnCo_2S_4$-$CoS_{1.097}$ cathodes at 2000 mA g^{-1} with a cut-off specific capacity of 1000 mAh g^{-1}; (b) Cycling performance of $MnCo_2S_4$-$CoS_{1.097}$ cathodes at 2000 mA g^{-1} under a specific capacity limit of 1000 mAh g^{-1}

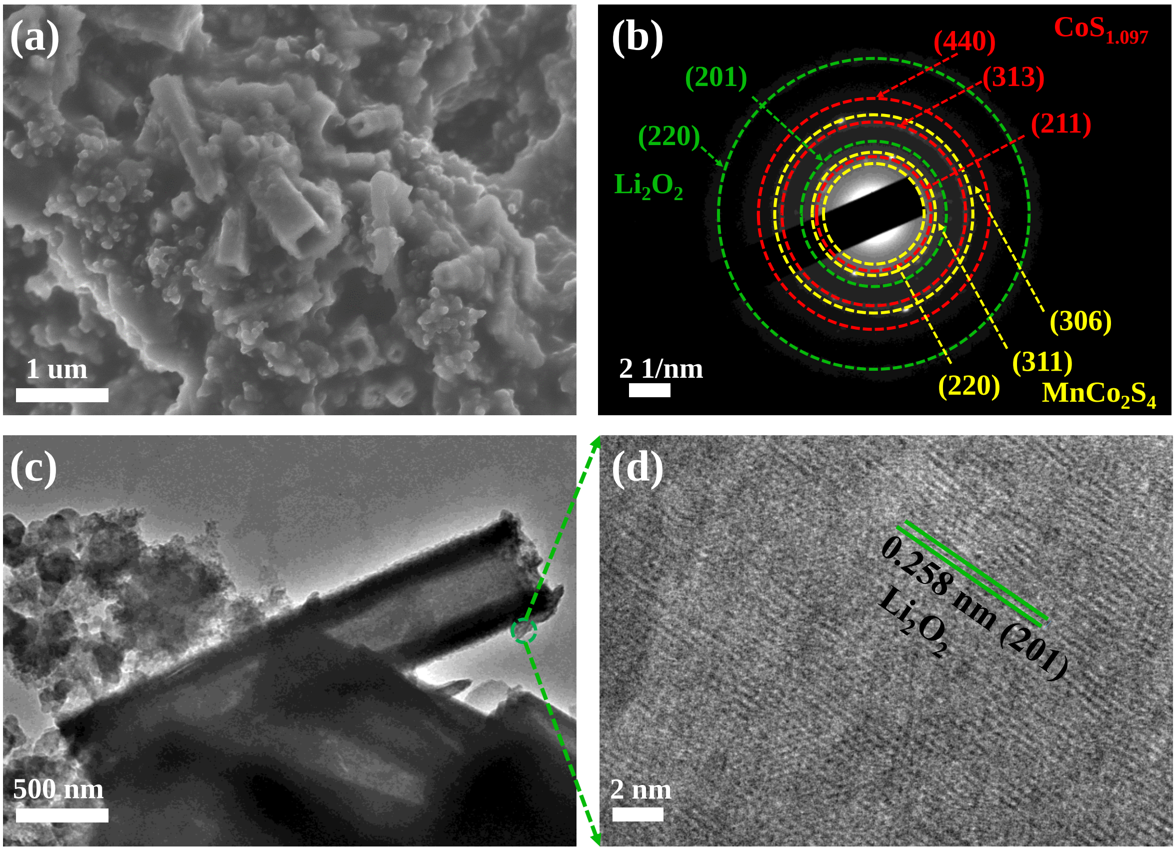

Figure S11. (a) SEM image (b) SAED pattern, (C) TEM image and (d) HRTEM image of $MnCo_2S_4$-$CoS_{1.097}$ cathodes after 1st discharging.
